# Determining Mammosphere-Forming Potential: Application of the Limiting Dilution Analysis

**DOI:** 10.1007/s10911-012-9258-0

**Published:** 2012-06-08

**Authors:** Lauren M. Rota, Deborah A. Lazzarino, Amber N. Ziegler, Derek LeRoith, Teresa L. Wood

**Affiliations:** 1 Department of Neurology & Neuroscience, NJMS Cancer Center H1200, New Jersey Medical School, University of Medicine and Dentistry of New Jersey, 205 S. Orange Ave., Newark, NJ 07101-1709 USA; 2 Division of Endocrinology, Diabetes and Bone Diseases, The Samuel Bronfman Department of Medicine, Mount Sinai School of Medicine, New York, NY 10029-6574 USA

**Keywords:** Mammosphere, Stem cell, Progenitor cell, Mammary, Breast, Tumorsphere, Cancer stem cell, Tumor initiating cell

## Abstract

Originally adapted from the neurosphere assay, the nonadherent mammosphere assay has been utilized to assess early progenitor/stem cell frequency in a given population of mammary epithelial cells. This method has also been used to measure the frequency of tumorsphere initiating cells in both primary mammary tumors as well as in tumor cell lines. Although, the mammosphere assay has been used extensively in the mammary gland field, a standard method of quantifying and analyzing sphere growth in this assay has remained undefined. Here, we discuss the use and benefit of using a limiting dilution analysis to quantify sphere-forming frequency in primary mammary epithelial cells grown in nonadherent conditions.

## Introduction

Stem cells, their developmental function, and their potential roles in disease, particularly cancer, have been under intense investigation over the past decade. The/mammary stem cell is self-renewing and capable of functionally reconstituting the entire mammary epithelial cell lineage [1–3]. Mammary stem cells are potential contenders for the origin and recurrence of some mammary tumors [1, 4]. Thus, cancer therapies targeting the subset of cancer stem cells or tumor initiating cells (TICs) may be most advantageous in treating these breast cancers and reducing the likelihood of recurrence [1].

A major hurdle in the mammary stem cell field has been to develop methods to identify and isolate the small subset of normal or cancer stem cells in epithelial tissue. Initial work to classify the stem cell population relied on lineage tracing and cell division activity in image studies of whole tissue and in serial transplantation analyses [5–7]. Subsequently, several groups published methods to identify cell surface markers of mammary gland stem and progenitor cells using flow cytometry and further linked these protein markers to in vivo transplantation stem cell activity [8–11]. More advances in the quest to identify and analyze mammary stem cells in vitro came when Dontu and colleagues described a nonadherent mammosphere assay (for review see [12] as well as Clarke and colleagues in this issue) which is an adaptation of the previously established neurosphere assay [13, 14]. In the original reports on mammospheres, human mammary epithelial cells were taken from reduction mammoplasties and grown over multiple passages in nonadherent conditions [12, 15]. These mammospheres maintained tripotentiality when allowed to differentiate in vitro by growth on either a collagen substratum or embedded in matrigel® in the presence of the pregnancy hormone, prolactin [15]. Mammospheres derived from reduction mammoplasties were found to be enriched for mammary epithelial stem cells which could produce successful outgrowths after transplantation of as few as 500 mammospheres into NOD/SCID mice [12]. Moreas and colleagues also showed that mammospheres derived from primary mouse mammary epithelial cells were capable of regenerating complete mammary epithelial trees upon transplantation [16].

The mammosphere assay has been widely utilized to measure in vitro stem/progenitor cell frequency in normal primary mammary epithelial cell preparations as well as frequency of cancer stem cells or TICs derived from malignant mammary tissue [12, 17]. Although the gold standard stem cell assay is the in vivo transplantation assay, first described by DeOme et al. [18], the mammosphere assay has given investigators an in vitro assay that is less time consuming and more cost effective then the in vivo transplantation assay [18, 19]. Although the mammosphere assay does not measure actual in vivo stem cell frequency, it can serve as a relative measure of stem and progenitor cell frequency in a mixed cell suspension, between varying experimental groups in vitro. Recently the stem cell field has been turning to a method popularized initially in immunology research, known as limiting dilution analysis (LDA). This quantitative approach incorporates the use of a limiting dilution cell culture assay and established statistical analysis. Several investigators have applied the quantitative LDA to evaluate the stem cell population in the neurosphere assay [20, 21]. Methods for quantifying the mammosphere assay vary within the field. Currently, similar to the neurosphere assay, sphere number is scored in a multi-sphere culture. However, this method is prone to subjectivity in sphere identification as well as culture artifacts (i.e. sphere aggregation/fusion). In the mammary gland field, many in vivo transplantation studies estimating stem cell frequency have successfully utilized limiting dilution to measure stem cell frequency [16, 22]. In this article, we discuss use of the quantitative LDA for analyzing stem/progenitor cell frequency in secondary and tertiary mammosphere cultures, and, hereafter, refer to this analysis as a “sphere limiting dilution analysis” (SLDA). Use of the SLDA has the advantage that it reduces investigator bias, can be used for both human and mouse mammary epithelial cell populations, and requires a smaller number of cells than the traditional sphere assay.

## Methods: Sphere Limiting Dilution Analysis

The SLDA operates on the assumption that a single cell can generate a sphere and that the variation within the experiment from well to well follows a Poisson distribution. (For a recent review see [23]). When conducting research on a mixed population of cells such as mammospheres we have found this analysis useful to evaluate the frequency of sphere formation as an indication of the presence of stem or early progenitor cells, and observe it to be consistent with sphere forming frequency derived from the traditional sphere counting approach.

**Figure 1:**
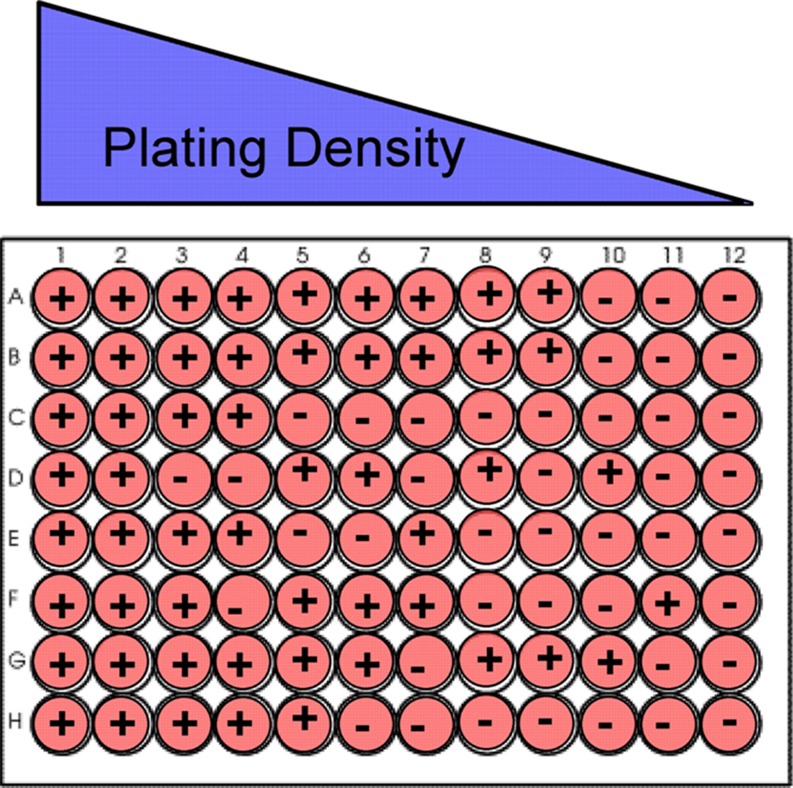
Schematic showing strategy for limiting dilution plating of dissociated epithelial cells in 96 well plate for SLDA. The “+” refers to a well containing one or more spheres and a “–” refers to a well without any spheres. The figure shows the cells being plated in a limiting dilution fashion across a 96-well plate. The amount of wells negative for sphere formation will increase as the plating density decreases as depicted

To perform the SLDA, the cells are plated into a 96 well plate over a range of densities from high to low (Fig. 1). The cells are then given a minimum of 7 days to form spheres. Plating density should be optimized, such that at the highest density, most wells will be positive for sphere formation, whereas the lowest density should have less then 1 cell per well, so the majority of wells are negative for sphere formation. The SLDA measures the probability of an event happening, i.e. sphere formation, and assumes that a percentage of wells at the lowest plating density will be non-responsive and not form spheres. Therefore, the line plotted from the experiment should pass through the origin (*x* = 0, *y* = 0) when graphing density versus probability of sphere formation or the fraction of non-responsive wells. To determine the probability of sphere formation, the wells are scored for the presence or absence of sphere growth. The natural log fraction of the negative wells (non-responding) is plotted on a linear scale versus the density. The density can be expressed as cells/cm^2^, cells/mL or cells/well [24]. For this review and for our experiments we choose cells/cm^2^ because it does not vary with volume of media in the well. A linear regression curve is then fit to the data points and the lower the density at which a population forms a sphere the higher its frequency for formation, by the equation

$$ \matrix{ {{\text{P}} = {1} - {{\text{e}}^{\text{mD}}}\left( {{\text{P}}\;{\text{is}}\;{\text{probability}},\;{\text{m}}\;{\text{is}}\;{\text{slope}}\;{\text{and}}\;{\text{D}}\;{\text{is}}\;{\text{density}}} \right),\;{\text{arranged}}} \\ {{\text{Ln}}\left( {{1} - {\text{P}}} \right) = {\text{mD}}\;{\text{where}}\;{\text{y}}\;{\text{is}}\;{\text{Ln}}\left( {{1} - {\text{P}}} \right)\;{\text{and}}\;{\text{x}}\;{\text{is}}\;{\text{D}}.} \\ }$$

According to the assumed Poisson distribution and as adopted from Lefkovits [24], the natural log of the fraction of non-responding wells is linearly proportional to the mean number of cells with sphere forming potential. When plotting data using the form of the equation shown here, the value of x at the *y* = −1 intercept corresponds to the frequency of sphere formation and represents 37 % non-responding wells. This value for different experimental groups can be compared to evaluate the frequency of sphere forming cells between varying conditions [24]. The frequency can be calculated at any point on the line by using the slope (frequency = 1/slope) or by using the *y* = −1 intercept (for detailed theory see Lefkovits [24]) however for ease of interpretation we prefer to use the *y* = −1 intercept for directly comparing precursor frequency of different populations.

Determining the frequency of sphere formation in culture assays can provide valuable information for interpreting the composition of the mixed population of starting cells. If used on cultures after treatment with a growth factor or receptor inhibitor, for example, the effects of the treatment on the percentage of sphere forming cells present in the population can be evaluated. Similarly, the consequence of genetic alterations on stem/progenitor cell number can be evaluated using SLDA. Similar to neurosphere cultures which enrich for mulitpotential progenitor cells over passage, the percentage of bipotential progenitor cells in nonadherent mammosphere cultures increases after several passages [12, 15]. Thus, using the SLDA on secondary or tertiary spheres allows one to study a more highly enriched bipotent progenitor population versus a more heterogeneous population in the primary spheres.

**Figure 2:**
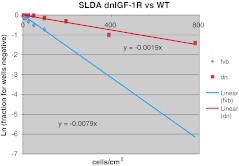
SLDA comparing stem cell frequency in tertiary spheres between dnhIGF-1R and FVB (WT) primary mammary epithelial cells grown in PRO-N media in 96 well ultra-low adherent plates (Corning). Plating density ranges from 1000 to 0.00012 cells/well, which was converted to cells/cm^2^ in this graph. Tertiary sphere forming frequency for dn-IGF-1R is 1 in 526 cells versus 1 in 127 cells for the wild type control. PRO-N media contained EGF/bFGF (20 ng/ml), bovine Insulin 25 μg/ml (Sigma), d-Biotin 10 ng/ml (Sigma), Progesterone 20 nM (Sigma), Putrescine 100 μM (Sigma), Selenium 5 ng/ml (Sigma), Apo-transferrin 50 μg/ml (Sigma), Gentamycin 50 μg/ml (Invitrogen-Gibco), Hydrocortisone 0.5 μg/ml (Sigma). Linear (fvb) and Linear (dn) indicate best fit line for linear regression analysis

We have used the SLDA to evaluate sphere formation in primary mammary epithelial cells (MECs) isolated from mammary glands expressing an MMTV-dominant negative insulin-like growth factor-1 receptor (dnIGF-1R) transgene to determine if defective IGF-1R signaling affected numbers of mammary stem/progenitor cells. Freshly isolated MECs were cultured as primary and then secondary mammospheres. The secondary spheres were dissociated with Accutase™ for 5 min at 37 °C and mechanically triturated until a single cell suspension was achieved. Cells were then plated at densities ranging from 1000 to 0.00012 cells (optimal range of cells for the SLDA in our mammosphere conditions; should be optimized for different cell populations and investigator conditions) across two 96 well plates in 200 μl of PRO-N media with 8 replicates for each dilution and then evaluated for tertiary sphere formation after 7 days in culture. We scored each well for the absence (−) or presence (+) of sphere growth to determine the fraction of negative wells (Fig. 1, schematic). The plot shows natural log transformation for the fraction of non-responding wells (y-axis) versus plating density (x-axis) (Fig. 2). As discussed, the probability of forming a sphere is determined by the x intercept (cell density) when *y* = −1. The sphere forming frequency of the dn-IGF-1R tertiary spheres (*y* = −1 intercept, *x* = 526), can be expressed as 1 in 526 cells capable of forming a tertiary sphere compared to the wild-type control cells that had a tertiary sphere forming frequency of 1 in 127 cells. Thus, we conclude that IGF-1R signaling is required for survival and/or sphere formation and may therefore be important for stem and/or progenitor cell growth. This result is consistent with sphere growth measures using a visual counting method in a multi-sphere culture.

## Discussion

Although, it is clear that mammospheres are enriched for multipotent cells, it remains somewhat ambiguous as to what passage number (i.e. primary, secondary, or tertiary) the spheres should be analyzed for the number of stem/multipotent progenitor cells in the population. It is generally accepted that number of spheres formed is equivalent to the combined number of stem and progenitors in a given population of mammary epithelial cells. The passage number (i.e. primary, secondary, etc.) and plating density, however, at which the mammospheres are analyzed has varied between investigators and could have an influence on the number of stem and progenitor cells present. Another limitation of the sphere assay relates to whether this assay properly identifies the frequency of in vivo quiescent stem cells as opposed to measuring cells that adapt or can act as a proliferating mammary stem cell in vitro. The validity of using the sphere assay as a functional test to measure frequency of in vivo stem cells was recently addressed in a review considering the correlation between neurosphere forming cells and number of neural stem cells in vivo [25]. Pastrana et al. concluded that the neurosphere assay does not properly identify numbers of quiescent neural stem cells in vivo. Furthermore, until these quiescent stem cells can be definitively elucidated using cell markers, the frequency of these cells in vivo will remain an enigma. The same caveats are applicable for the mammosphere assay; it is still unclear whether the mammosphere assay truly identifies quiescent stem cells in the mammary gland. Also, multi-potential progenitor cells can give rise to sphere growth as well as stem cells. In addition, although stem cell growth and survival factors such as wnt and hedgehog have been identified, optimal growth conditions have not been standardized for mammosphere growth; therefore, the mammosphere assay is likely not a true measure of physiological stem cell frequency. Mammosphere cultures, like neurosphere cultures, however, do enrich for epithelial cells that behave like mammary stem cells in vitro and are capable of forming all three lineages when transplanted into cleared fat pads [16].

Within the limitations of the mammosphere assay, by standardizing methods in quantifying the mammosphere assay using the SLDA, it is possible to reduce bias between experiments and between investigators. Furthermore, using the SLDA to quantify mammospheres in secondary and subsequent passages, due to their known enrichment for multipotent cells, will allow for greater confidence in interpretation that the calculated number of sphere forming cells correlates with number of multipotential progenitors or stem cells. The use of the SLDA on primary mammospheres should be avoided if possible when measuring multipotent cell frequency because primary cultures are more heterogenous then are secondary and tertiary sphere cultures as discussed by Dontu and colleagues (2005), however, in circumstances where a genetic alteration or treatment condition prevents subsequent passaging of mammospheres, it would still be useful to assess primary sphere forming frequency. An example of this was seen with constitutive activation of smoothened in mammary epithelial cells [16]. Because the SLDA takes multiple plating densities (from clonal to higher densities) into account, it allows for a more comprehensive method of quantifying sphere formation and sphere size in a given population of cells. Sphere size may be a reflection of a mitogenic response to specific treatment conditions and progenitor expansion, however, due to sphere aggregation, emphasis has been placed on analyzing sphere size at clonal densities [25]. In the SLDA, sphere size can accurately be evaluated at the lower densities (i.e. 1 ≤ cell per well). In summary, the SLDA is an advantageous tool in quantifying sphere forming frequency in a population of mammary epithelial cells. The SLDA does not require a large amount of cells so can be utilized when sample quantities are limited. Due to low density plating, it also reduces problems of cell aggregation. Finally, the SLDA method reduces investigator bias and can lead to a more standard method for quantifying stem/progenitor cell frequency in mammospheres.

## Abbreviations

TIC: Tumor initiating cells
NOD/SCID, LDA: Limiting dilution analysis
SLDA: Sphere limiting dilution analysis
MECs: Mammary epithelial cells
MMTV: Mouse mammary tumor virus promoter
dnIGF-1R: Dominant-negative insulin like growth factor type 1 receptor
WT: Wild type
EGF: Epidermal growth factor
bFGF: Basic fibroblast growth factor

## References

[1] Reya T, Morrison SJ, Clarke MF, Weissman IL (2001). Stem cells, cancer, and cancer stem cells. Nature.

[2] Molyneux G, Regan J, Smalley MJ (2007). Mammary stem cells and breast cancer. Cell Mol Life Sci.

[3] Spike BT, Engle DD, Lin JC, Cheung SK, La J, Wahl GM (2012). A mammary stem cell population identified and characterized in late embryogenesis reveals similarities to human breast cancer. Cell Stem Cell.

[4] Al-Hajj M, Wicha MS, Benito-Hernandez A, Morrison SJ, Clarke MF (2003). Prospective identification of tumorigenic breast cancer cells. Proc Natl Acad Sci U S A.

[5] Chepko G, Smith GH (1999). Mammary epithelial stem cells: our current understanding. J Mammary Gland Biol Neoplasia.

[6] Kenney NJ, Smith GH, Lawrence E, Barrett JC, Salomon DS (2001). Identification of stem cell units in the terminal end bud and duct of the mouse mammary gland. J Biomed Biotechnol.

[7] Kordon EC, Smith GH (1998). An entire functional mammary gland may comprise the progeny from a single cell. Development.

[8] Stingl J, Eirew P, Ricketson I, Shackleton M, Vaillant F, Choi D (2006). Purification and unique properties of mammary epithelial stem cells. Nature.

[9] Stingl J, Eaves CJ, Zandieh I, Emerman JT (2001). Characterization of bipotent mammary epithelial progenitor cells in normal adult human breast tissue. Breast Cancer Res Treat.

[10] Shackleton M, Vaillant F, Simpson KJ, Stingl J, Smyth GK, Asselin-Labat ML (2006). Generation of a functional mammary gland from a single stem cell. Nature.

[11] Sleeman KE, Kendrick H, Ashworth A, Isacke CM, Smalley MJ (2006). CD24 staining of mouse mammary gland cells defines luminal epithelial, myoepithelial/basal and non-epithelial cells. Breast Cancer Res.

[12] Dontu G, Wicha MS (2005). Survival of mammary stem cells in suspension culture: implications for stem cell biology and neoplasia. J Mammary Gland Biol Neoplasia.

[13] Reynolds BA, Weiss S (1996). Clonal and population analyses demonstrate that an EGF-responsive mammalian embryonic CNS precursor is a stem cell. Dev Biol.

[14] Reynolds BA, Weiss S (1992). Generation of neurons and astrocytes from isolated cells of the adult mammalian central nervous system. Science.

[15] Dontu G, Abdallah WM, Foley JM, Jackson KW, Clarke MF, Kawamura MJ (2003). In vitro propagation and transcriptional profiling of human mammary stem/progenitor cells. Genes Dev.

[16] Moraes RC, Zhang X, Harrington N, Fung JY, Wu MF, Hilsenbeck SG (2007). Constitutive activation of smoothened (SMO) in mammary glands of transgenic mice leads to increased proliferation, altered differentiation and ductal dysplasia. Development.

[17] Liu JC, Deng T, Lehal RS, Kim J, Zacksenhaus E (2007). Identification of tumorsphere- and tumor-initiating cells in HER2/Neu-induced mammary tumors. Cancer Res.

[18] Deome KB, Faulkin LJ, Bern HA, Blair PB (1959). Development of mammary tumors from hyperplastic alveolar nodules transplanted into gland-free mammary fat pads of female C3H mice. Cancer Res.

[19] Dontu G, Al-Hajj M, Abdallah WM, Clarke MF, Wicha MS (2003). Stem cells in normal breast development and breast cancer. Cell Prolif.

[20] Bull ND, Bartlett PF (2005). The adult mouse hippocampal progenitor is neurogenic but not a stem cell. J Neurosci.

[21] Ziegler AN, Schneider JS, Qin M, Tyler WA, Pintar JE, Fraidenraich D, et al. IGF-II promotes stemness of neural restricted precursors. Stem Cells. 2012: (in press), *Indicates co-senior authors. 10.1002/stem.1095 PMC5581406 22593020

[22] Lindeman GJ, Visvader JE, Smalley MJ, Eaves CJ. The future of mammary stem cell biology: the power of in vivo transplants. Breast Cancer Res. 2008;10(3):402; author reply 3. doi:10.1186/bcr1986. 10.1186/bcr1986 PMC2481484 18522763

[23] Hu Y, Smyth GK (2009). ELDA: extreme limiting dilution analysis for comparing depleted and enriched populations in stem cell and other assays. J Immunol Methods.

[24] Lefkovits I, Waldmann H (1999). Limiting dilution analysis of cells of the immune system.

[25] Pastrana E, Silva-Vargas V, Doetsch F (2012). Eyes wide open: a critical review of sphere-formation as an assay for stem cells. Cell Stem Cell.

